# Relating to the end of life through advance care planning: Expectations and experiences of people with dementia and their family caregivers

**DOI:** 10.1177/14713012211066370

**Published:** 2022-01-03

**Authors:** Natashe Lemos Dekker, Sascha R Bolt

**Affiliations:** Institute of Cultural Anthropology and Development Sociology, 450192 Leiden University, Leiden, the Netherlands; TRANZO Tilburg School of Social and Behavioral Sciences, Tilburg University;, Tilburg, the Netherlands Department of Health Services Research, CAPHRI School for Public Health and Primary Care, Living Lab in Ageing and Long-Term Care, 5211 Maastricht University, Maastricht, the Netherlands

**Keywords:** End-of-life, palliative care, advance care planning, future, control, qualitative research

## Abstract

**Background:**

Dementia is widely considered a progressive condition associated with changes in cognitive capacities, which promotes the idea that people with dementia need to anticipate end-of-life care preferences. There is a growing body of interventions meant to support advance care planning (ACP) for people with dementia and their families. However, a deeper understanding of their experiences and expectations regarding planning the end of life is needed to inform ethically sound and person-centered ACP. This study explores how end-of-life care planning for people with dementia is experienced and perceived in practice.

**Methods:**

We conducted a secondary analysis of qualitative data from in-depth interviews and ethnographic fieldwork. Participants included people with dementia living in the community or in nursing homes, family caregivers, and professional caregivers. During reflective sessions, the researchers moved back and forth between their original data and overarching topics related to ACP. Both performed deductive coding to filter relevant information from their data. Ongoing discussions allowed for the fine-tuning of themes.

**Results:**

End-of-life care planning for people with dementia can encompass a paradox of control. While it may foster a sense of being in control, it may also feel like relinquishing control over future situations. It raises concerns regarding responsibility, as present, former and future wishes may not align. Family caregivers are often responsible for concrete end-of-life decision-making, prompting them to weigh previous wishes against present situations. Further, people with dementia may wish to focus on the present and distance themselves from the future.

**Discussion:**

Advance decisions about end-of-life care are often decontextualized and people may find it difficult to oversee their future conditions and preferences. The widely recognized purpose of ACP to exert control over end-of-life care may need revision to match the needs of people with dementia while they are coping with their condition and an uncertain future.

## Introduction

Advance care planning (ACP) is a communication process that allows patients and their families to discuss care goals and future care preferences with professional caregivers (Rietjens et al., 2017). ACP may involve but is not limited to the documentation of care preferences in advance directives. Various definitions of ACP exist, and most entail recurrent conversations, as care preferences may change throughout the course of an illness (IOM, 2015; Rietjens et al., 2017). ACP is one of the recommended key domains for palliative care for people with dementia (van der Steen et al., 2014). Due to the terminal nature of dementia and the associated degeneration of cognitive functions, including decision-making capacity, there has been a growing interest in end-of-life care planning as a part of ACP for people with dementia specifically (Dixon et al., 2018). Cognitive decline is a common argument for timely initiation of end-of-life conversations so that persons with dementia are still capable of expressing their own wishes and preferences (Brighton & Bristowe, 2016; Harrison Dening et al, 2019). ACP theoretically involves discussions about holistic future care needs, more general personal values, and life goals (Rietjens et al., 2017). Yet, in practice a prominent part of ACP still focuses on end-of-life care planning and writing advance directives, especially in nursing homes where this is often part of the submission process or periodic evaluation (Shaw et al., 2020; Thoresen et al., 2016).

In light of this, we approach end-of-life care planning, as a part of ACP, in this article as a particular future-oriented vehicle of care. It is a way of anticipating, through which people with dementia are asked to envision their preferences in circumstances that might occur in the future, and to make decisions and act upon this in the present. In this process, family members often take on a prominent role as caregivers who often become proxy decision-makers for the person with dementia in the future (Lord, Livingston, & Cooper, 2015). Therefore, definitions of ACP include family involvement (IOM, 2015; Rietjens et al., 2017). However, people with dementia and their proxies may hold different views on the development of the condition and relate differently to the future (Hellström & Torres, 2016). Views on desired end-of-life care may thus also differ between them (Ayalon et al, 2012; Harrison Dening et al, 2013; Harrison Dening et al, 2016; Poole et al., 2018).

Recent studies show that, particularly in the early stages, people with dementia and their families may lack readiness to talk about the future or the end of life (Poole et al., 2018) and that in order to achieve potentially beneficial effects of ACP, its conduct necessitates patients’ readiness (Zwakman et al., 2018). Studies that examined perspectives of people with dementia themselves demonstrate mixed findings regarding their willingness to establish advance care plans. Thinking about end-of-life care can be complex, fear inducing, and confronting (Dickinson et al., 2013; Sellars et al., 2019). Furthermore, people with dementia may question the potential benefits of discussing future care preferences, or they may prefer informal, undocumented conversations about care (Dickinson et al., 2013; Poole et al., 2018). Some studies suggest that people with dementia and their families may be more willing to engage in ACP when they are more acceptant of the condition and its terminality (Sellars et al., 2019). Others suggest that conversations about clinical scenarios and end-of-life care goals with people with dementia and their families may foster acceptance and enable informed proxy decision-making in the future (Givens et al., 2018). Orsulic-Jeras and colleagues have proposed strategies to initiate early care-related discussions among people with dementia and their caregivers with the aim of improving decision-making in later stages (Orsulic-Jeras et al., 2019). Moreover, Ryan and colleagues propose that the “right time” for initiating ACP conversations balances between individuals’ decreasing mental capacities and their insight into the consequences of having dementia (Ryan et al, 2017). This presumes dementia to be a linear trajectory in which such a moment can be clearly identified. However, in practice, dementia fluctuates and is difficult to predict or oversee, making the “right time” for (advance) decision-making often unclear and difficult to assess for healthcare professionals (Robinson et al., 2013), as well as for people with dementia themselves and their families (Lemos Dekker, 2020).

Despite its suggested importance, the actual engagement of people with dementia in ACP is limited (de Boer et al, 2012). Worldwide, reported proportions vary from 3% to 39% (Sellars et al., 2019). Existing models of ACP do not translate well to people with dementia, being poorly adapted to the practical and ethical challenges related to the individuals’ cognitive decline (Robinson et al., 2013; van den Block, 2019). The notion that ACP should be person-centered and that it requires a person’s readiness to talk is well-described (Wendrich-van Dael et al., 2020; Zwakman et al., 2018). Reluctance of individuals to engage in ACP is a widespread phenomenon, which raises ethical questions about different agendas with regard to end-of-life care planning and whether the “ACP policy” is actually and indisputably in the patient’s best interest (Russell, 2014; Ryan et al., 2017). The suggested early initiation of end-of-life care planning, when persons with dementia and their family caregivers are trying to cope with the condition and an uncertain future, also raises ethical concerns and the question to what extent this supports everyday living with dementia and facing an ambiguous future and end of life.

A recent review found only four studies that investigated strategies to increase participation of people with dementia in ACP, of which none included their own perspective (Bryant et al., 2019). The exclusion of people with dementia from research is a widespread concern, as it prevents their voices from being heard and limits understanding their experiences (Fletcher, 2020; Perfect et al., 2019). To inform ethically sound ACP practice, we need a deeper understanding of the befalling of end-of-life care planning in practice and on the experiences and attitudes from people with dementia themselves and their families. This study aims to explore how end-of-life care planning as a part of ACP for people with dementia unfolds in practice and how this relates to the experiences and expectations of people with dementia and their family caregivers.

## Methods

This study combines the results of three previously conducted qualitative studies on end-of-life and palliative care in dementia. The combined analysis was carried out adopting a naturalistic interpretative approach (Bhattacherjee, 2012), which involved providing a narrative “thick description” of the phenomenon based on the primary ethnography, naturalistic inquiry, and qualitative descriptive research. The study participants were people with dementia, family caregivers, and professional caregivers. Participants with dementia lived either in the community or in nursing homes in the Netherlands. The motivation for combined analysis is the synergies that we found in the findings in relation to ACP, which was not the primary focus of the initial, independent analyses. Together, these studies comprise a broad population of people with dementia, their families, and their professional caregivers in different phases of the dementia trajectory and in different long-term care settings. Further, the joint analysis of the study findings provides an encompassing view of the manifestations of ACP across different regions in the Netherlands.

## Study designs and data collection

NLD conducted 18 months of ethnographic fieldwork in different regions of the Netherlands (North Holland and Zeeland) (Table 1, study 1). The research design combined participant observations and informal conversations in nursing homes and in-depth interviews in home settings. The participants were people with dementia; their family members; and professional caregivers, including physicians, chaplains, and nursing staff. The observations and informal conversations were recorded in ethnographic field notes; the semi-structured interviews were audio-recorded and transcribed verbatim. This study aimed at understanding the experiences of people with dementia, their family members, and professional caregivers, in striving toward a good end of life.

**Table 1.** Overview of the individual studies.

Study #	Design	Methods	Analysis	Participants	Setting
1	Ethnographic study	Observation, informal conversations, and in-depth interviews	Grounded theory approach and data-driven coding	People with dementia, family caregivers, and professional caregivers	Nursing homes and home settings
2	Qualitative descriptive	Semi-structured, in-depth interviews	Thematic analysis, theory, and data-driven coding	Bereaved family caregivers (*n* = 32)	Community (home) and nursing homes
3	Qualitative descriptive	Semi-structured, in-depth interviews	Thematic analysis and data-driven coding	People with dementia (*n* = 18)	Community (home)

SB conducted two studies (Table 1, studies 2 and 3) in a southern region of the Netherlands (South-Limburg), both using semi-structured in-depth interviews. Study 2 focused on family caregivers’ experiences with care at the end of life of a relative with dementia, either at home or in a nursing home. Study 3 focused on people with dementia who lived in the community and their perspectives on their future, end-of-life, and on being cared for by others. Interviews from both these studies (2 and 3) were audio-recorded and transcribed clean verbatim. The studies were part of a larger research project entitled DEDICATED (Desired Dementia Care Towards End of Life). The overall aim of DEDICATED is to improve palliative care for people with dementia, by empowering nursing staff and improving interprofessional and transmural collaboration.

Ethical approval for conducting study 1 was obtained from the Ethical Committee of the Amsterdam Institute for Social Science Research (AISSR). The Medical Ethical Committee METC Z approved study 2 (METCZ20180026) and study 3 (METCZ20180085). In all three studies, written or verbal informed consent was obtained from the study participants or, when appropriate, legal representatives. Participants in the primary studies also gave consent for the use of their data in secondary studies related to the topic of palliative care and dementia. The authors did not share personal data of research participants with one another. Any data shared between us were anonymized transcripts, which were coded by the primary researcher of that study, which is in line with contemporary data protection regulations.

## Analysis

The current paper describes a secondary thematic analysis of a combination of different sources of qualitative data, using principles of directed content analysis (Hsieh & Shannon, 2005). The researchers, both trained in conducting qualitative research, have backgrounds in anthropology and clinical neuropsychology. Both researchers had previously analyzed their separate data independently of each other. In this secondary analysis, the focus shifted to experiences, attitudes, and observations related to end-of-life planning as a standalone activity or as an integrated activity in ACP and shared decision-making. Original data sources included transcripts from individual in-depth interviews and field notes from ethnographic work. During several initial meetings, the researchers discussed their findings in relation to planning for end-of-life care, looking for resemblances and contradictions in the data. During these in-depth discussions about the data and primary analysis, the researchers first formulated key concepts and recurring elements. These concepts formed the basis for analyzing transcripts and selecting text fragments with more pertinent content. The researchers then coded the original data sources using a new coding scheme based on the overarching concepts. The authors regularly discussed their coding to allow cross-validation in the analysis. The researchers discussed the coded content to interpret the data and to generate an overview of recurring themes and interpretations. They then further fine-tuned the themes throughout the mutual analysis process to make sure all relevant content was covered.

Both researchers had used reflexive bracketing in their original studies to become aware of their role as interviewers, observers, and analysts (Tufford & Newman, 2012). For instance, this meant having reflective discussions with dementia care professionals, informal caregivers, and members of the research team about the study topic and methods. This supported their understanding of others’ and their own preexisting assumptions and potential biases, which may rise from personal characteristics (e.g., age, gender, and belief systems) and professional roles (e.g., academic roles, areas of expertise, and professional background). The researchers discussed the data inspection during frequent reflective meetings, which allowed them to develop a shared understanding of their data and linkages between them. Triangulation in the data collection methods increased the credibility of the joint analysis.

## Results

This section describes four overarching themes derived from the combination of the studies. The themes illustrate the unfolding of ACP in practice and the fundamental issues that may arise in ACP for people with dementia, their family caregivers, and their professional caregivers. The themes are (1) a paradox of control, (2) present and future wishes, (3) a matter of responsibility, and (4) avoiding end-of-life conversations.

### The paradox of control

Being able to establish wishes and preferences in advance, before becoming unable to express them verbally or in writing due to dementia, was sometimes experienced as reassuring and provided a sense of control. One participant with dementia recalled how he had witnessed the decline of a friend, who also had dementia and who had lived in a nursing home. Not wishing this for himself, he stated:

Then I said, that will never happen to me, I am sure. Then someone else says “easy for you to say that now, but…” and I say “no, this is my decision, and it is final, you can count on that.” (…) I want it to be clear and in writing before the time comes. (Person with dementia, study 3)

At the same time, participants did not always perceive their written directives and care preferences as conclusive. For some, documented wishes still involved a sense of uncertainty:

That has been documented by the GP. The [case manager] knows how I feel about it. There is a note, ‘do not resuscitate’ in the home care file. (…) it is a notarial documentation right, but you know, it is not a certainty, but oh well, it is written down. (Person with dementia, study 3)

Paradoxically, the idea that ACP allows for the person with dementia to maintain control over their own later stages of life was at times refuted by a sense that making decisions in advance meant relinquishing control. ACP could evoke the fear that fixing care plans for future needs would not allow them to deviate from these plans should their preferences change. As one dementia case manager explained:

People are also afraid that if they record their wishes, that this might be acted upon even without them wanting it any more. People tend to be very hesitant towards that. On the one hand it [to record wishes] gives some reassurance, but on the other they also find it scary. (Care professional, study 1)

This became apparent, for example, in one nursing home where it was common practice to place a red adhesive label on the care folder of residents with a non-resuscitation plan. One resident, who had secured her wish not to be resuscitated in an advance care plan, was nevertheless hesitant toward the red sticker. The topic was discussed during several meetings with her family members and professional caregivers, but she held off on a decision for three years. Eventually, during one of these conversations, she asked:

If I break a leg, and I have a red sticker, will I still be cared for? (Person with dementia, study 1)

It became clear that she had assumed that the red sticker not only applied to resuscitation but also to other aspects of care and worried that she would no longer be cared for in the event of an emergency. This situation reflects that different interpretations and miscommunication regarding advance directives may further complicate the matter of maintaining and relinquishing control in the context of dementia.

One of the interview participants with dementia experienced mixed feelings regarding the documentation of his wishes for future care. He was aware of the inevitable decline of his capacities, which elicited anxiety:

What if it gets out of hand, the, what do I call it, “further collapsing of the brain tissue”. Well, that will be an unimaginably difficult situation to bear, to cope with. (Person with dementia, study 3)

He did not want to talk too much about his future, as he found this very confronting. At the same time, thoughts about the future and the end of life occupied him and he was frightened by the idea of deterioration. He had told his GP, informally, that he did not want to live through severe decay. Yet, he was hesitant to document his wishes. He and his wife noted that he would have to explicate and document this before his cognitive functioning would decline, which they thought could mean that it was “too late”:

Yes, so, we did make sort of an arrangement, but we did not put it in writing. And we did not discuss it sufficiently to put it all in writing. I don’t know, I don’t know. I, if I would say yes, write it down, write something down. It would be a major step for me to do that. (Person with dementia, study 3)

### Present and future wishes

While ACP is considered important in the context of dementia in order to express end-of-life wishes before becoming unable to do so, this is also complicated as preferences may change as the condition progresses. Friction may occur between previously established preferences and care plans, and what the person with dementia wants in the present. We found that ideas about future and end-of-life care were often influenced by negative images surrounding dementia (e.g., that the end of life with dementia would be dehumanizing). This is important to consider when weighing previous and present wishes and goals of care, as negative imaginaries may not accurately reflect how people with advanced dementia actually perceive their situation. Several participants with dementia referred to their “future selves” in terms of helplessness and insignificance, which influenced their thoughts about preferred future care:

Look, if I will be completely demented, clearly that would be the end of the story. I don’t know where I would end up in that case, but, yeah, that seems like a threatening exit to me. (Person with dementia, study 3)

Furthermore, especially relatives of the person with dementia often found it difficult to decide whether to adhere to the established preferences or the person’s present wishes. For example, one woman with dementia living at home had stated that she wished not to be resuscitated or sent to a hospital (study 1). However, when she fell and broke her hip, her daughter was initially uncertain about what to do but eventually called an ambulance, despite her mother’s previous preferences. She did not want her mother to be in pain and therefore decided to have her admitted to the hospital. The wish not to be hospitalized had been made explicit. However, such previously established wishes are made without knowing the circumstances in which these might take place and thus may not fit with the needs when the actual situation arises. In another case, the person with dementia had explicated that she *did* want to go to the hospital, and her two daughters disagreed when a choice had to be made:

So, when the end approached, you have to make choices. Go to the hospital? My sister thinks about what other people would say. I don’t. I was like, this is what she would have wanted, so this is what will be done, period. (Family caregiver, study 2)

In addition, family caregivers may have difficulties to understand the consequences of deciding for or against specific (medical) treatments. They may not be able to envision how a treatment will affect their relative with dementia. This sometimes led to difficult discussions, according to a healthcare worker in one of the nursing homes:

When someone has a cardiac arrest, and you try to resuscitate them, the chances of pulling them through and in what state, the prospects for this are so bad. Still, some people find it difficult to accept the non-resuscitation policy we have within the psychogeriatric unit. And then still they cannot make that decision, saying, “well, she is still doing well now” or “no, I cannot say that so definitely yet” (Care professional, study 1).

In some cases, family members and professional caregivers held different views as to which directives to follow. For example, a nursing home resident refused to drink or eat and wanted to be left alone. His daughters asked the nursing staff if something could be done about this, but they replied that there was nothing they could do as long as he continued to refuse. The daughters disagreed and stated:

But he is demented. That means he is mentally incompetent, doesn’t it? (Family caregiver, study 1)

The nursing staff, however, repeated that:

We have to listen to him as long as he can indicate what he wants. (Care professional, study 1)

This encounter between family and care staff reflects the difficulty of knowing how to interpret the present wishes of the person with dementia versus previously established preferences as well as concerns with their well-being. Even as family members were often uncertain how to weigh the past and present wishes of the person with dementia, they were often in a position responsible for important decisions.

### A matter of responsibility

Family caregivers of people with dementia in the studies often took on a responsible role in making care and treatment decisions on behalf of their relative. Some of the family caregivers did not recall having had formal ACP conversations, or at least not with the person with dementia being present:

We did, well, not with her present, but we did say at her admission that there would be no need to resuscitate her. (Family caregiver, study 2)

Nonetheless, most family caregivers referred to formal or informal conversations about topics related to end-of-life care. For instance, topics related to resuscitation and hospitalization had often been discussed with care professionals, especially at admission into a nursing home:

We knew that beforehand, my mother had already said, no resuscitation, then we stated this and she agreed to that, no more fuzz to her body. (…) Yes, that was documented. (Family caregiver, study 2)

Although some family caregivers reported not to have discussed end-of-life preferences with the person with dementia, most of them said they knew the person’s wishes in a broad sense. “Knowing” these wishes sometimes meant that the person with dementia had actually declared them at some point. However, “knowing” could also refer to a protective sense of knowing what is best for the person in absence of a formal declaration. One family caregiver, who was a nurse herself, stated that it was impossible to talk about the end of life with her husband with dementia. She would care for him at home in a way she felt suited him, and she did not want to hand over this care to a nursing home, as was often suggested by the home care staff that supported her:

Yes, of course we discussed this [nursing home admission], but I simply wouldn’t have it. Really, I would not have it, even though we discussed more and more frequently that, if this situation would continue, nursing home admission would be needed. We never discussed this with him though, but he would not have wanted it. (Family caregiver, study 2)

Persons with dementia themselves often expressed trust in their family or formal caregivers to make the best decision on their behalf. Some preferred to rely on the other’s assessment of what is best, rather than discussing their own wishes for the end of life. This way, responsibility is willingly placed in the hands of trusted others. Some expressed they did not feel confident to make decisions due to a lack of knowledge and thought a medical professional could better estimate what was best in the situation:

I think physicians are very good at telling up front how a patient will come out of anesthesia, or something, because it is a certain process. (Person with dementia, study 3)

Knowing or assuming a relative’s wishes for the end of life sometimes raised challenging situations for family caregivers who felt responsible to comply with those wishes. Some family caregivers experienced doubt, confusion, or ethically challenging situations when trying to make the best decision on behalf of their relative. Knowing the person’s explicit wishes helped in some, but not all, situations:

Chest pain, I know all about it, that is far from comfortable and then you would actually want to get help, to get rid of it. But there was a living will, no more life-prolonging treatments (…). So, we were actually in doubt at that point. (Family caregiver, study 2)

Decision-making can also be ethically challenging, having to decide on life and death. This can also emotionally weigh on family caregivers:

Then she [nurse] said, well, shall we start the IV? And I said, how? Start the IV? But then he will die. Yes, she replied, but we discussed this, right? I said yes, we did discuss it, but- (…) And then it started to hit me, Jesus, he is going to die. (Family caregiver, study 2)

### Avoiding end-of-life conversations

Persons with dementia themselves generally preferred to focus on the present and on what they were still capable of doing, instead of dwelling on the future or the end of life. Some of them recognized this as an avoidant way of coping with an unsettling future:

You think about it, but at some point you do hold it off. It’s just, now I can still do this and do that. But the core of it, what it is in fact all about, of course you avoid that as long as possible. (Person with dementia, study 3)

Some others stated that they were in general not interested to discuss their wishes for care at the end of life. Several interviewed family caregivers also indicated that the end of life was not something that was discussed openly with the person with dementia:

My mother never wanted to talk about that. She never said, like, I would want it in this or that way, or end-of-life treatment in this or that moment. It was not a topic for discussion, no. (Family caregiver, study 2)

Not having end-of-life conversations had different underlying reasons, such as a feeling that discussing care preferences would not make a difference in the end or the idea that thinking about the future would be too distressing for oneself:

I do not want to think it through that far. No, then you only make it more difficult for yourself. (Person with dementia, study 3)

Some family caregivers also indicated that their relative with dementia did not actively talk about the end of life because they were focusing on the present:

She lived in the here and now, purely. Some people make plans and I don’t know what else, but I think that just did not occur to her at all. (Family caregiver, study 2)

Other reasons for avoiding end-of-life conversations were that participants thought it was too early to discuss such topics, that the person with dementia did not feel the need to discuss such issues before the actual situation arose, or that they did not want to discuss their own death:

If I have to suffer, there is still enough time to talk about that. For now, just let me be. (Person with dementia, study 3)

Most participants with dementia did not explicitly regard death itself as particularly threatening or burdensome. A more prominent threat was deteriorating functioning and decreasing independency due to their condition. Rather, death was, by most, regarded as an inevitable, natural event. Some individuals with dementia, as well as some former caregivers who were more skeptical about discussing end-of-life care, referred to the end of life and death as something that cannot be controlled and planned. This idea fostered a sense of acceptance and resignation with regard to the end of life, which also meant focusing on the present:

Well, of course this [end of life] comes to mind sometimes, but I do not want to wrap my head around it. I am living now, and how it will be in the future, I will find out then. (Person with dementia, study 3)

## Discussion

We have illustrated how the process of end-of-life care planning for people with dementia unfolds in practice. We described how family caregivers, professional caregivers, and people with dementia themselves perceive and experience discussing end-of-life care, and how this may relate to decision-making. The views of people with dementia are essential to developing an understanding of the potential value, limitations, and drawbacks of end-of-life care planning as a part of ACP. Our findings suggest that the ACP process for people with dementia can entail a paradox of control and discrepancies between current and future wishes, and poses challenges related to responsibility. Further, it could undermine a person’s wish to refrain from discussing the future or the end of life, and to focus on the present.

Definitions of ACP tend to be broader than medical and end-of-life care planning, and involve the aim of providing holistic care that is in line with a patient’s own preferences, values, and goals (Rietjens et al., 2017). ACP can serve varying populations of patients with chronic and life-limiting conditions, by promoting shared decision-making in setting goals of palliative and end-of-life care (Brinkman-Stoppelenburg, 2014). It also has the potential to reduce unwanted hospital admissions, and to increase the use of palliative care services or hospice care, and it may lead to more tailored care. Nonetheless, our studies suggest that in practice, ACP was often still regarded and experienced as medical end-of-life care planning by people with dementia, family caregivers, and professionals. This is in line with previous studies in the context of nursing homes (Thoresen et al., 2016) and beyond (Shaw et al., 2020). Other studies have demonstrated that the uptake of ACP is generally low, not only in people with dementia (Sellars et al., 2019), but also in other patient populations such as in people with heart failure, COPD, and Parkinson’s disease (Walter et al., 2019; Williams et al., 2020). Different patient populations may raise distinct barriers to ACP. In dementia, the uncertain prognosis, unrecognized terminal nature, and decreasing decision-making capacity are common and specific barriers (De Vleminck et al., 2014; Robinson et al., 2013; Sellars et al., 2019). In this study, we exposed challenges raised by end-of-life care planning which may partly explain the low uptake of ACP in routine dementia care practice.

We identified a paradox of control that occurs when discussing and documenting future medical care. This may give people with dementia a sense of “being in control” over their future, while at the same time, securing one’s wishes in advance may feel like relinquishing control over future situations. ACP was originally developed to facilitate autonomous decision-making regarding future treatment options by enabling people who may become incapacitated to establish their wishes in advance (Fleuren et al, 2020). Underlying this is an assumption that choice on the part of the patient leads to better future medical care (Borgstrom, 2015). However, the exact circumstances of these future events are unknown in advance. Zivkovic refers to “temporal dissonance” in documenting advance directives as a disconnection that occurs when placing oneself ahead of time, not knowing how the situation and one’s perspective toward the situation may have changed by then (Zivkovic, 2018). End-of-life care planning asks individuals to establish their wishes while they are unable to oversee the variety of aspects that would complicate decision-making once the situation actually presents itself (Borgstrom, 2015; Sudore & Fried, 2010). Hence, advance medical decisions are often decontextualized and may overlook the changeability of conditions, circumstances, and perceptions.

The issue of decontextualization in end-of-life care planning relates to the understanding that people often find it difficult to predict their own attitudes, preferences, and behaviors in future situations, or how they will cope with disability or illness (Halpern & Arnold, 2008; Loewenstein, 2005; Pols & Limburg, 2016). Previous studies underline that people with dementia and older people in general feel uncertain about what their future will bring and how they will cope in more advanced stages of their decline (Dickinson et al., 2013; Hill et al., 2007; Seymour et al., 2004). Cognitive problems, such as memory loss and difficulties in communication and decision-making, combined with the unpredictable and uncertain course of the condition, make ACP in dementia challenging (van den Block, 2019). Although the decline of cognitive functioning is a common argument for early ACP conversations, it also poses a serious challenge for people with dementia to oversee their own future preferences. Establishing medical care preferences in advance could raise feelings of “foreclosing” or limiting future care options, especially since people are expected to make decisions about treatment or its withdrawal, which precludes alternatives, as well as the possibility to learn to live with certain circumstances they did not consider manageable beforehand (Weidemann, 2012; Zivkovic, 2018). Yet, some decisions can be made explicitly to foreclose unwanted futures (Lemos Dekker, 2020). The implication in ACP is that people with more advanced dementia must rely on the estimation of their “old self” with regard to medical care preferences. In a similar way, proxy decision-makers may face ethical challenges in decision-making when weighing former wishes against a person’s current state and preferences (de Boer et al., 2010).

In the context of dementia and the associated cognitive decline, ACP is considered to facilitate the person to exert control over their future medical care and treatments (Harrison Dening et al., 2019). However, the pivotal role of the family in ACP, both at its initiation and in situational decision-making, draws into question to what extent this presupposed key advantage of end-of-life care planning actually holds. As our results show, family members took much responsibility in ACP. They took on the responsibility to formulate the wishes and care plans for their relative with dementia, regardless of whether this had or had not been discussed before. We observed that formal ACP conversations with healthcare professionals often occurred at admission to the nursing home and that these mainly revolved around medical care planning. At this point, the person with dementia was often unable to articulate his or her wishes, which meant that their family caregivers were primarily involved on their behalf. Moreover, the lack of context for medical decisions when made in advance means that their implications and validity have to be re-evaluated by family and professional caregivers in a given situation. This often involved reconsidering the established wishes in light of the situation at hand, at which point they had to decide whether to stick to, or deviate from, the formulated wishes. In practice, we saw that professional and family caregivers would sometimes have to deviate from established wishes in order to provide good care. For family caregivers and people with dementia themselves, making end-of-life care decisions or establishing such wishes in advance, may be complicated by a lack of detailed knowledge, for instance, about prognosis, treatment options, or legal aspects of ACP (Dickinson et al., 2013; Kaufman, 2006; Sellars et al., 2019). This further complicates end-of-life care planning because it involves decisions that are, in principle, difficult to oversee without the required, specialized knowledge.

Often, ACP literature surpasses the question if the need for medical end-of-life care planning as a part of ACP is universally shared and whether it carries equal value and benefits for all who face life-threatening illnesses or are at the end of life. Various studies do point out that ACP requires willingness and readiness from patients and their family caregivers (Wendrich-van Dael et al., 2020; Zwakman et al., 2018). Nonetheless, the emphasis on medical treatments and control, predominantly in Western countries, may influence how individuals perceive and engage with ACP. When unwilling to engage in ACP, this is often framed as pathological (Russell, 2014) or as being in denial. Moreover, people may face stigmatization if they do not wish to plan their medical end-of-life care or if they make choices that are considered to be wrong by healthcare professionals or others (Barnard, 2002). Our study demonstrates that establishing wishes and preferences for medical treatment in advance does not provide the same level of reassurance for everyone, and that, for some, it may be confronting and fearsome. The particular dynamic of relinquishing control in order to maintain it can also be frightening and cause uncertainty. In many cases, family members are also uncertain whether to follow or deviate from previously established wishes, as both options can be ethically challenging. These findings raise ethical questions regarding the desirability of end-of-life care planning in its current form for people with dementia and their families.

## Conclusion

In this study, we assessed the experiences of people with dementia, their family members, and care professionals, as they engaged in, or refrained from, end-of-life care planning in practice. While having end-of-life care conversations or establishing one’s wishes for future medical care in written form can give some people with dementia a sense of control over an uncertain, and in many cases fearful, future, others may experience end-of-life care planning itself as frightening and uncertain.

Even when a person’s wishes have been made explicit, the actual decision-making with regard to treatment at the end of life is in many cases relegated to family and professional caregivers. Further, a central concern in end-of-life care planning is that medical decisions are made in a decontextualized manner, that is, relating to a situation that cannot be fully known in advance. Also, people’s outlook on changing circumstances may vary over time. Some people with dementia were unwilling to engage in ACP altogether, which demonstrates the need to create room for people who wish not to engage in care planning, without labeling this as pathological. The widely recognized purpose of ACP to exert control over end-of-life care may need revision to match the needs of people with dementia while they are coping with their condition and an uncertain future.
